# Lessons for conservation management: Monitoring temporal changes in genetic diversity of Cape mountain zebra (*Equus zebra zebra*)

**DOI:** 10.1371/journal.pone.0220331

**Published:** 2019-07-31

**Authors:** Antoinette Kotzé, Rae M. Smith, Yoshan Moodley, Gordon Luikart, Coral Birss, Anna M. Van Wyk, J. Paul Grobler, Desiré L. Dalton

**Affiliations:** 1 National Zoological Garden, South African National Biodiversity Institute, Pretoria, South Africa; 2 Department of Genetics, University of the Free State, Bloemfontein, South Africa; 3 Department of Zoology, University of Venda, Thohoyandou, Republic of South Africa; 4 Flathead Lake Biological Station, Division of Biological Sciences, University of Montana, Missoula, Montana, United States of America; 5 Wildlife Program, Fish and Wildlife Genomics Group, College of Forestry and Conservation, University of Montana, Missoula, Montana, United States of America; 6 CapeNature, Gatesville, South Africa; Macquarie University, AUSTRALIA

## Abstract

The Cape mountain zebra (*Equus zebra zebra*) is a subspecies of mountain zebra endemic to South Africa. The Cape mountain zebra experienced near extinction in the early 1900’s and their numbers have since recovered to more than 4,800 individuals. However, there are still threats to their long-term persistence. A previous study reported that Cape mountain zebra had low genetic diversity in three relict populations and that urgent conservation management actions were needed to mitigate the risk of further loss. As these suggestions went largely unheeded, we undertook the present study, fifteen years later to determine the impact of management on genetic diversity in three key populations. Our results show a substantial loss of heterozygosity across the Cape mountain zebra populations studied. The most severe losses occurred at De Hoop Nature Reserve where expected heterozygosity reduced by 22.85% from 0.385 to 0.297. This is alarming, as the De Hoop Nature Reserve was previously identified as the most genetically diverse population owing to its founders originating from two of the three remaining relict stocks. Furthermore, we observed a complete loss of multiple private alleles from all populations, and a related reduction in genetic structure across the subspecies. These losses could lead to inbreeding depression and reduce the evolutionary potential of the Cape mountain zebra. We recommend immediate implementation of evidence-based genetic management and monitoring to prevent further losses, which could jeopardise the long term survival of Cape mountain zebra, especially in the face of habitat and climate change and emerging diseases.

## Introduction

The Cape mountain zebra (CMZ, *Equus zebra zebra*) provides a useful case study to help understand and advance the usefulness of genetic tools and monitoring for biodiversity conservation. The Cape mountain zebra is a subspecies of mountain zebra that is endemic to South Africa. The subspecies is listed as Vulnerable on the International Union for Conservation of Nature (IUCN) Red List [1] and on Appendix II by the Convention on International Trade in Endangered Species (CITES; [2, 3]). Historically, Cape mountain zebra had a widespread distribution in the mountainous Fynbos, Karoo, and grassland regions of the Western Cape, the Eastern Cape and portions of the Northern Cape Provinces of South Africa [4, 5, 6]. However, by the 1950’s, as a direct result of hunting pressure and habitat loss, CMZ experienced a 90% reduction in its geographic distribution and were reduced to less than 50 individuals in a few relict populations, inhabiting the most inaccessible parts of the subspecies’ range [1, 7, 8]. Only three relict populations survived to the present day, and these are found in the Mountain Zebra National Park (MZNP) located near the town of Cradock in the Eastern Cape, and Kammanassie National Reserve (KNR) and Gamkaberg National Reserve (GNR) in the Klein Karoo region of the Western Cape.

Following historical decline, CMZ numbers have since recovered to an estimated 2,650 individuals within the historical range [9,6], due mainly to dedicated conservation efforts by the South African National Parks (SANParks), who are the custodians of the MZNP. The low proportion of habitat that contains palatable grasses such as *Themeda trinadra* is one of the main limiting factors to CMZ growth in many of the current populations [10, 11, 12]. Therefore, SANParks employed a range expansion strategy, where “excess” MZNP stock was used to seed several other populations within the former CMZ range including: Baviaanskloof Nature Reserve, Karoo National Park, Camdeboo National Park, Tankwa Karoo National Park, Bontebok National Park, Oorlogskloof Nature Reserve, DeHoop Nature Reserve (DHNR); and outside its natural distribution range including: Addo Elephant National Park, Table Mountain National Park, West Coast National Park, Commando Drift Nature Reserve, Tsolwana Nature Reserve and Gariep Dam Nature Reserve. In addition, approximately 1,500 CMZ are reported to occur on private reserves [8]. The populations of MZNP-derived CMZ in South Africa have steadily increased and the estimated number of mature individuals in protected areas has exceeded the threshold of 1,000 for more than five years, resulting in a regional IUCN Red Listing of Least Concern [6].

In contrast, to the SANParks approach, CapeNature, the authority managing the remaining relict populations at KNR and GNR, has historically opted for a more conservative approach, citing low population numbers and lower growth rates as reasons not to remove animals from their reserves to establish populations elsewhere. The only exception to this rule was the population at DHNR, founded from a mix of MZNP and KNR individuals in the 1960s and 1970s. Thus, despite KNR and GNR containing a large proportion of the CMZ’s historic genetic variation [7], they remain isolated and at critically low numbers.

Genetic factors likely influencing the persistence of CMZ have been previously reported. Moodley and Harley [7] indicated that individual CMZ populations exhibited low genetic variation. The three relic CMZ sub-populations (MZNP, KNR and GNR) were inbred with the lowest microsatellite heterozygosity being identified in KNR (H_e_ = 0.239). In sharp contrast, the only known mixed population at DHNR had the highest genetic diversity (H_e_ = 0.380, [7]). However, the overall genetic variation in the metapopulation (populations from National South African Reserves) was considered moderate because substantial remnant allelic variation existed in the subspecies [7]. Inbred populations of CMZ with low genetic diversity show an increased incidence of tumours due to equine sarcoidosis which is reported to manifest from complex interactions between the aetiologic agent, the environment and the host genome. [13, 14, 15]. In addition, the two relict CMZ populations (GNR and KNR) further exhibited inflated genetic differentiation due to genetic drift and inbreeding effects resulting from lack of dispersal [7].

To manage and monitor the evolutionary potential of the CMZ metapopulation, a Biodiversity Management Plan (BMP) for this species in South Africa was developed by various stakeholders. The purpose of the BMP is to ensure the long term survival of CMZ in nature, using a strategy underpinned by specific goals and objectives aimed at addressing the threats faced by this subspecies, such as population fragmentation, disease, inbreeding, hybridization with plains zebra and habitat loss [6, 9]. It was suggested by several authors [6, 7, 8, 15, 16, 17] that mixing of aboriginal populations is required to further reduce genetic diversity losses, especially considering that only populations descending from MZNP stock are experiencing high population growth whereas KNR and GNR are not.

However, it was recommended that introductions into either KNR and/or GNR populations be avoided due to the observed population structure, and mixing should only be considered at alternative locations which would be monitored. These plans were hindered, however, by recent ecological studies that suggested CMZ numbers in the GNR were too low to risk removal of individuals to seed mixed populations [18, 19], despite the high diversity reported for the mixed DHNR population. Currently, an estimated 40 mountain zebra are being removed from the MZNP for the purpose of re-establishing a breeding herd within the historical range as well as stocking private reserves with animals, both within and outside the natural distribution range. To date, Cape mountain zebra occur in more than 75 localities, including over 30 national parks. Population sizes are estimated to vary between 4 and 1,191 individuals, with the largest population found in the MZNP. The average annual population increase for the subspecies over the period 2009–2015 was 11% [8].

Although the CMZ metapopulation continues to grow, it is yet to be determined whether management strategies have affected genetic diversity over time. Thus, this study aims to investigate temporal changes in genetic diversity in three key CMZ subpopulations by comparing present day (2015–2016) levels with those of samples collected from 1999 to 2001 [7]. This represents a time span of approximately 15 years or approximately 1.5 generations [20, 6]. Genetic diversity within populations can only be expected to increase through gene flow between relict stocks (e.g., as in DHNR) or new mutations, with the latter considered inconsequential in the timescale involved. Natural selection is unlikely to increase diversity except at individual loci under balancing selection. Given that our survey is only across a single generation, and there has been no gene flow between relict stocks during this time, we do not expect diversity to have increased over the study period.

However, diversity may be maintained in larger populations experiencing rapid growth. Therefore, we expect that within one generation, populations with larger size and high growth rates (e.g., MZNP) should maintain diversity, whereas populations with lower growth (e.g., DHNR), and smaller size (KNR), would be expected to show a loss in genetic diversity. Furthermore, changes in genetic diversity such as loss of heterozygosity and rare alleles may also have consequences for how populations are structured relative to each other. The loss of shared alleles from populations would be expected to inflate genetic structure (differentiation), as seen in Moodley and Harley [7], whereas a loss of private alleles would reduce genetic differentiation, making populations appear more similar. The results of this study will be used to inform management strategies employed by the CMZ BMP, by providing additional data on population diversity and differentiation.

## Methods

### Ethical approval and sample collection

Ethical approval was obtained from the Research Ethics and Scientific Committee (RESC) of the National Zoological Garden, South African National Biodiversity Institute (NZG SANBI, NZG/RES/P17/19), as well as the Animal Ethics Committee of University of the Free State (UFS-AED2017/0011). Permission was also obtained from the Department Agriculture Forestry and Fisheries of South Africa under Section 20 of the Animal Disease Act 1984 with (Ref: 12/11/1/1/8). The CMZ were chemically immobilised by helicopter. The dosages of sedation and reversal drugs as well as administration were carried out by a qualified, licensed veterinarian, registered with the South African Veterinary Council. Whole blood samples (5 ml) were collected by a qualified veterinarian from the MZNP (n = 75) and DHNR (n = 27) in 2016 and four samples were obtained from KNR in 2015. In addition, this study includes samples from MZNP (n = 12), DHNR (n = 15) and KNR (n = 9) collected between 1999 and 2001 [7]. Thus, a total of 142 samples were analysed. Samples were stored at -20°C in the Biobank of the NZG, SANBI until used.

### Molecular methods

We extracted DNA using the Quick-DNA Universal kit (Zymo Research) following the manufacturer’s protocol for blood. We selected 14 cross-species microsatellite markers (AHT21, UCDEQ505, HTG3, HTG7, HTG9, HTG11, HTG14, HTG15, LEX20, LEX52, TKY273, VHL47, HMB1 and COR014) used in the study conducted by Moodley and Harley, (2005). Polymerase Chain Reaction (PCR) amplification was conducted in a 12.5 μl reaction volume consisting of Ampliqon *Taq* DNA Polymerase Master Mix RED, forward and reverse primers (0.5 μM each), and 50 ng genomic DNA template. The conditions for PCR amplification were as follows: 5 min at 95°C denaturation, 30 cycles for 30 sec at 95°C, 30 sec at 55–60°C (depending on the marker amplified, S1 Table), and 30 sec at 72°C, followed by extension at 72°C for 40 min in a T100 Thermal Cycler (Bio-Rad Laboratories, Inc.). PCR products were run against a Genescan 500 LIZ internal size standard on an ABI 3130 Genetic Analyzer (Applied Biosystems Inc.). Samples were genotyped using GeneMapper v. 4.0 software (Applied Biosystems Inc.).

### Genetic variation

MICRO-CHECKER software [21] was used to detect possible genotyping errors, allele dropout, and null alleles. Allelic richness (A_r_), was estimated correcting for sample size through rarefaction using HP-RARE v. June-6-2006 [22]. Allele Frequencies, observed Heterozygosity (H_o_) and expected heterozygosity (H_e_) and number of private alleles per population was calculated using GenAlEx 6.5 [23, 24]. To determine the significance of changes (H_e_ or A_r_) between the two time periods, a one tailed pairwise T-test (α = 0.05 and α = 0.1) was performed with a null hypothesis that no loss in diversity has occurred. Deviations from expected Hardy-Weinberg (HW) proportions were tested (Markov Chain length of 105 and 100,000 dememorization steps). We also tested for gametic disequilibrium between all pairs of loci using the exact test described by Guo and Thompson [25] in GenAlEx 6.5.

### Bottleneck simulations

The programme Bottleneck version 1.2.02 [26] was used to detect evidence of recent population bottlenecks. This programme measures significant differences between the measured expected heterozygosity (H_e_; i.e., gene diversity) and the theoretical expected heterozygosity assuming mutation-drift equilibrium (H_eq_) given the sample size and observed number of alleles (A). This test identifies populations that have recently undergone a decline in effective population size (N_e_), resulting in a heterozygosity excess and deficit of rare alleles [27]. It was suggested that, through testing for bottlenecks using heterozygosity excess, a population size reductions of ~50 N_e_, occurring approximately 25–250 generations ago can be detected [27]. Previously recommended parameters for microsatellite data, using the two-phase mutation model, were used [28, 27]. This model accommodates for a small proportion of multiple-step mutations, with most mutations being a single step change in allele length. We used two different mutation models including 95% and 80% single-step mutations (SMM) with a two-phase model variance of 12% for a total of 10,000 iterations [29]. Heterozygosity excess was tested for using the Wilcoxon sign-rank test (Significance at α = 0.05) [30]. Allelic frequency, mode-shift deviation from the L-shaped distribution was examined, which was to corroborate detection of a recent bottleneck event [31].

### Effective population size (N_e_) estimation

The two-sample temporal method [32, 33, 34] was applied to estimate the variance effective population size (N_eV_). This method assumes temporal changes in allele frequencies are caused solely by genetic drift, based on the Wright Fisher model [35, 36]. The standardized variance in allele frequency was calculated using two moment based *F*-statistic estimators, namely *F*s [37] and *F*c [34] under the model that assumes animals sampled will contribute to future generations. This was implemented using the program NeEstimator v2.1 [38]. This analysis was performed using population samples from MZNP which consisted of adults and foals. The KNR and DHNR were omitted from this analysis due to the small sample size and the recent admixture.

### Genetic structure

Since changes in genetic diversity can also affect genetic structure, we determined the relative structure (differentiation) among our three sampled populations at the time periods 1999–2001 and 2015–2016 using three methods. First, we used the Principal Component Analysis (PCA), which is a multivariate method using K-means clustering, and implemented in the R package Adegenet version 2.1.1 [39]. We then used the model based Bayesian clustering algorithm in STRUCTURE version 2.3.4 [40], which determines the most probable number of populations and assigns individuals to their most likely population of origin.

We ran STRUCTURE with the following models: admixture model with both correlated and independent allele frequencies and no admixture model with correlated and independent allele frequencies. Each of the models were run without prior population information for ten replicates each with K = 1–10, with a run-length of 700,000 Markov Chain Monte Carlo repetitions, following a burn-in period of 200,000 iterations. The ten values for the estimated log-likelihood (ln(*Pr*(*X*|*K*)) were averaged across runs and posterior probabilities were calculated. The *K* with the greatest increase in posterior probability (ΔK, [41]) was identified as the optimum number of sub-populations using STRUCTURE HARVESTER [42]. The membership coefficient matrices (Q-matrices) of replicate runs for the optimum number of sub-populations was combined using CLUMPP version 1.1.2 [43] with the FullSearch algorithm and G′ pairwise matrix similarity statistics. The results were visualized using DISTRUCT version 1.1 [44]. Lastly, we used an F_st_-based hierarchical analysis of molecular variance (AMOVA, [45]) to estimate how genetic diversity was partitioned between and within the MZNP and KNR populations for both time periods (Arlequin 3.5; [46]). We excluded the DHNR population for this particular analysis as it is descended from a mix of MZNP and KNR.

## Results

### Genetic variation in different populations and time periods

Deviations from HW proportions, following Bonferroni correction [47], were only observed in two loci from two populations namely: COR014 (MZNP, DHNR, 2015–2016) and UCDEQ505 (DHNR, 2015–2016). One locus, COR014, showed evidence of null alleles in the MZNP population at both time periods. The locus, UCDEQ505 showed evidence of null alleles in one population at one time period (2015–2016) according to the MICRO-CHECKER results (S2 Table). Thus, all further analysis was performed with and without the marker COR14. The KNR 2015–2016 population was not tested for null-alleles due to insufficient sample size. Significant gametic disequilibrium was not observed between loci in any population. The markers, HTG15, LEX52 and HTG03 were monomorphic in all CMZ samples for both time periods and were omitted from further analysis. Analysis of microsatellite data identified low to moderate genetic diversity (1999–2001: H_e_ = 0.511 and 2015–2016: H_e_ = 0.338) in CMZ populations from both time periods (S3 Table) compared to those reported for plains zebra (*Equus quagga*; H_e_ ranged from 0.71 to 0.80) [48]. In the 1999–2001 population, the A_r_ was 2.069, the average H_o_ was 0.327 (range of 0.115 to 0.531) and the average H_e_ was 0.503 (range of 0.356 to 0.720). In the 2015–2016 population, the A_r_ was 1.86, the average H_o_ was 0.274 (range of 0.061 to 0.621) and the average H_e_ was 0.337 (range of 0.119 to 0.627).

### Genetic diversity and effective size (N_e_): temporal changes within populations

**Fig 1:**
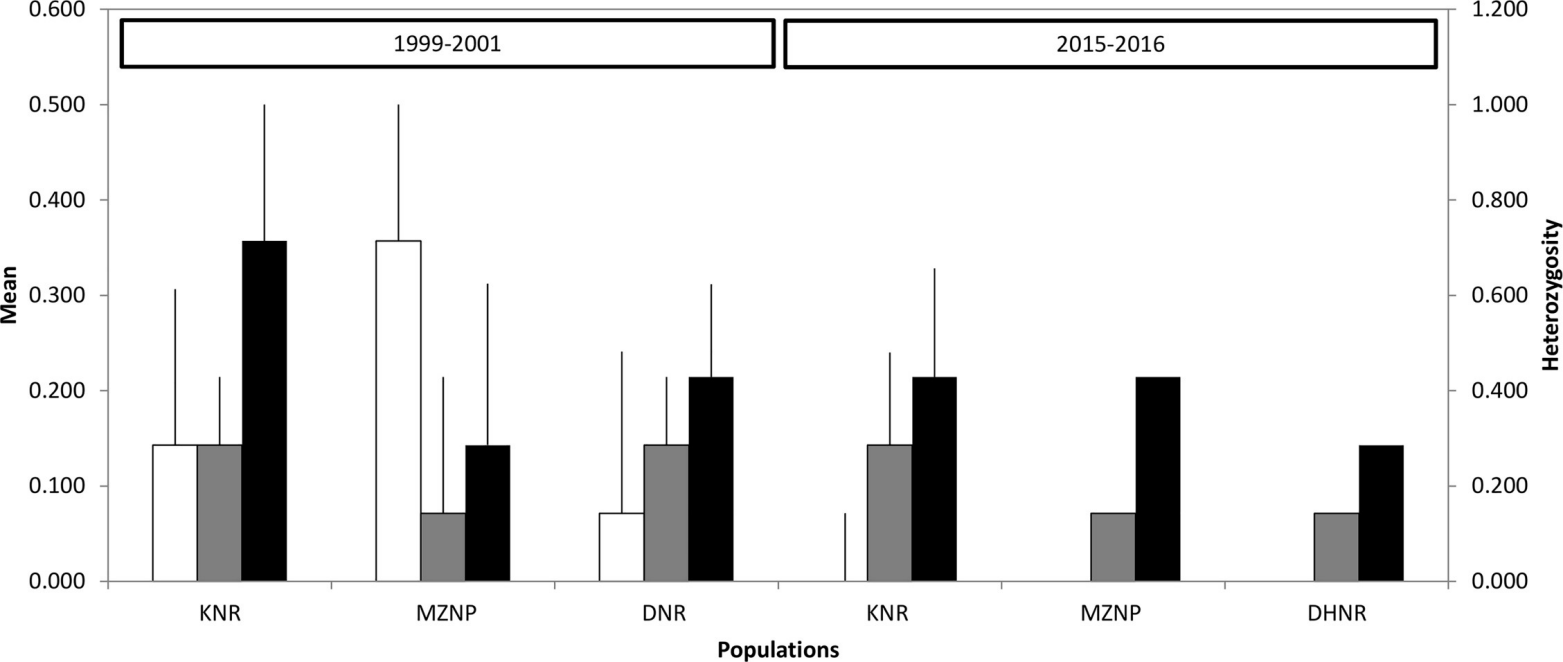
Loss of private alleles (red) in a single generation from 1999/2001 to 2015/2016 in each of three Cape mountain zebra populations. White indicates the frequency of private alleles unique to a population, grey indicates the frequency of locally common alleles (frequency of < = 5%), black indicates the frequency of locally common alleles found in 50% or fewer populations (frequency of > = 5%). Mountain zebra National Park (MZNP), De Hoop Nature Reserve (DHNR) and Kammanassie Nature Reserve (KNR) for the temporal periods 1999–2001 and 2015–2016.

Analysis per population indicated that the highest heterozygosity and A_r_ was observed for the DHNR population at both temporal periods compared to the MZNP and KNR populations (Table 1). Heterozygosity within each reserve (KNR, DHNR and MZNP) declined between temporal sampling periods (Table 1). A decline in genetic diversity was observed for DHNR in A_r_, which decreased from 2.35 to 2.1 between 2015–2016 and 1999–2001. H_e_ declined from 0.385 to 0.297 during this same ~16 year time period. In the MZNP population, the mean A_r_ declined from 1.65 to 1.53 and H_e_ was reduced from 0.264 to 0.230. In the KNR population A_r_ decreased from 1.59 to 1.53 and H_e_ declined from 0.258 to 0.230 (Table 1). However, only the decline in H_e_ in DHNR was observed to be statistically significant (p > 0.05). The number of private alleles for KNR, MZNP and DHNR in 1999–2001 was 0.143, 0.357 and 0.071 respectively, whereas the frequency of private alleles in these populations in 2015–2016 was 0.0 (Fig 1). The temporal variance estimates of N_e_ ranged from 1.7 to 6 when performing F_c_ analysis and varied from 1.7 to 18.2 when performing F_s_ analysis (Table 2). Analysis where the locus COR014 was removed was similar for F_c_ analysis but differed for F_s_ (1.6 to 31.2, Table 2).

**Table 1:** Genetic diversity at the two time points for the Cape mountain zebra in the three reserves, Mountain zebra National Park (MZNP), De Hoop Nature Reserve (DHNR) and Kammanassie Nature Reserve (KNR). Population genetic diversity over for two periods spanning around 16 years (1999–2001 to 2015–2016) was evaluated based on allelic richness (A_r_), observed heterozygosity (H_o_) as well as unbiased expected Heterozygosity (H_e_) and fixation (F_IS_) across 11 polymorphic microsatellite loci. Detection of recent bottlenecks were tested by identifying whether Mountain Zebra National Park had significant levels of heterozygous excess using the Two Phase Mutation model (Wilcoxon P_TPM_ and P_SMM_) and then confirmed by checking allelic mode shift at mutation-drift equilibrium (A_DIST_). * and bold text = significant temporal change, *p* < 0.05. ^1^indicates estimates of A_r_ are corrected for sample size through rarefaction in HP-RARE using the smallest number of gene copies per population (MZNP = 14, DHNR = 24, KNR = 4) and ^2^indicates were analysis was not applicable, since sample size was too small for KNR and in DHNR, the population is an admixture of MZNP and KNR.

Reserve	Period	Sample size	A_r_^1^ (SE)	H_o_(SE)	H_e_(SE)	F_IS_(SE)	Wilcoxon P_TPM_	Wilcoxon P_SMM_	A_DIST_
MZNP	1999–2001	12	2.27(0.33)	0.167(0.07)	0.264(0.09)	0.383(0.09)	0.922	0.922	NA^2^
	2015–2016	75	1.95(0.33)	0.221(0.07)	0.230(0.07)	0.076(0.05)	0.615	0.688	Normal
DHNR	1999–2001	15	2.35(0.21)	0.394(0.07)	**0.385(0.05)**	-0.011(0.10)	NA^2^	NA^2^	NA^2^
	2015–2016	27	2.1(0.21)	0.266(0.07)	0.297(0.07)*	0.042(0.10)	NA^2^	NA^2^	NA^2^
KNR	1999–2001	9	1.59(0.12)	0.205(0.07)	0.258(0.07)	0.126(0.13)	NA^2^	NA^2^	NA^2^
	2015–2016	4	1.53(0.12)	0.218(0.10)	0.230(0.09)	-0.080(0.12)	NA^2^	NA^2^	NA^2^

**Table 2:** Estimated effective population sizes for the Mountain zebra National Park (MZNP) using the temporal method. The two analyses used were the F_c_ method (Nei and Tajima, 1981) and the F_s_ method (Jorde and Ryman, 2007) using the Program NeEstmator ver. 2.1 (Do et al., 2014). P_crit_ = the criterion for excluding rare alleles if the frequency of rare alleles and less than the P_crit_ value they are excluded, GI = the Generation interval, N_e_ = the estimated effective population size, Min and Max = confidence interval values and CV = the Coefficient of variation.

MZNP	Analysis with marker COR14				Analysis without marker COR14			
	Fc		Fs		Fc		Fs	
	P_crit_ = 0,05	P_crit_ = 0,02	P_crit_ = 0,05	P_crit_ = 0,02	P_crit_ = 0,05	P_crit_ = 0,02	P_crit_ = 0,05	P_crit_ = 0,02
**GI**	1	1	1	1	1	1	1	1
**N**_**e**_	3.2	3.4	3.2	3.2	3.1	3.3	3.1	3.2
**CI 95% (Min—Max)**	1.7–5.8	1.9–6	1.7–18.2	1.8–15.1	1.5–6.2	1.7–6.2	1.6–31.2	1.7–24.1

### Bottleneck tests

The bottleneck tests were only carried out on the MZNP, for the two temporal periods. Similar results were obtained for both 80% and 95% SMM (Table 1). The DHNR population was not tested as the heterozygote excess method assumes that no recent admixture has taken place in KNR, and the sample size was too low. No significant heterozygote excess was detected for MZNP (p > 0.10).

### Genetic structure

**Fig 2:**
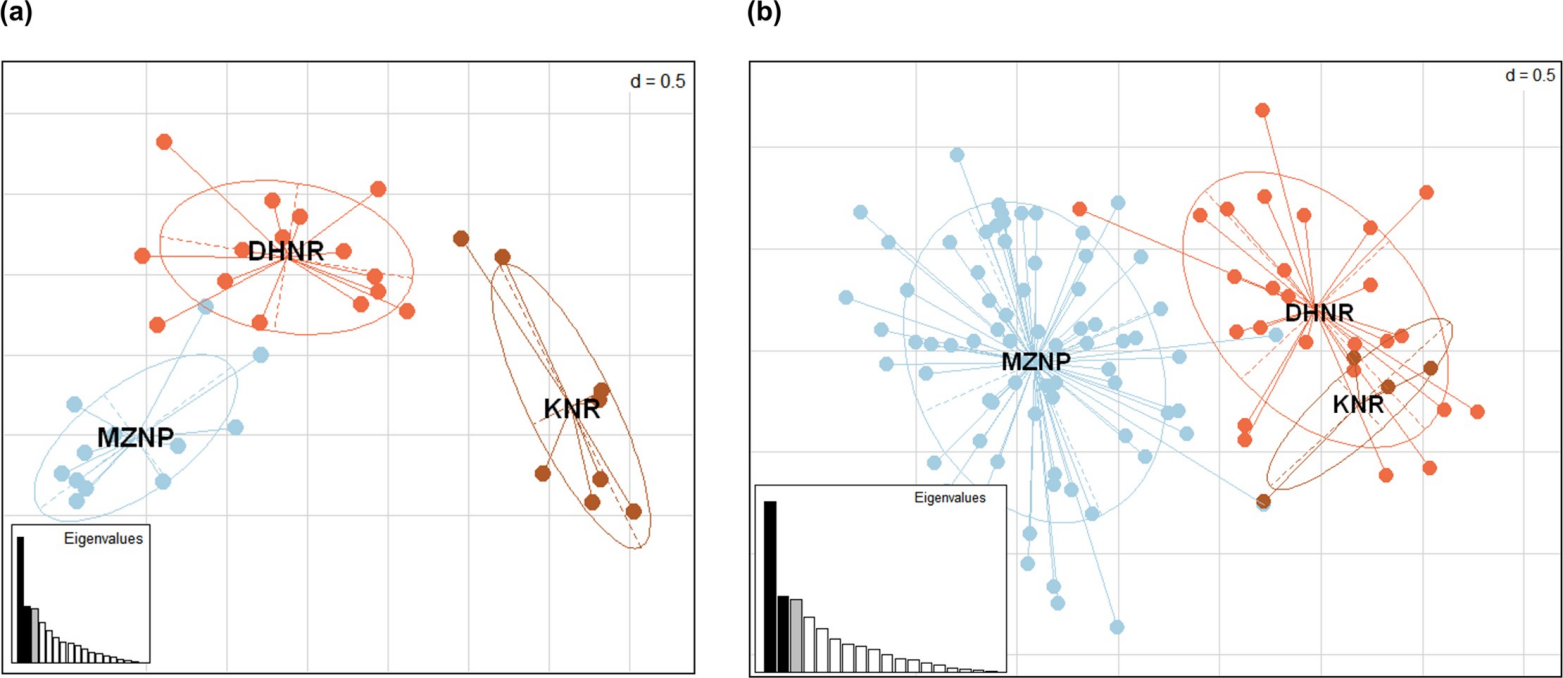
Principal Component analysis for three Cape mountain zebra populations at two temporal periods. (a) 1999–2001 and (b) 2015–2016, for the populations Mountain Zebra National Park (MZNP), De Hoop Nature Reserve (DHNR) and Kammanassie Nature Reserve (KNR).

**Fig 3:**
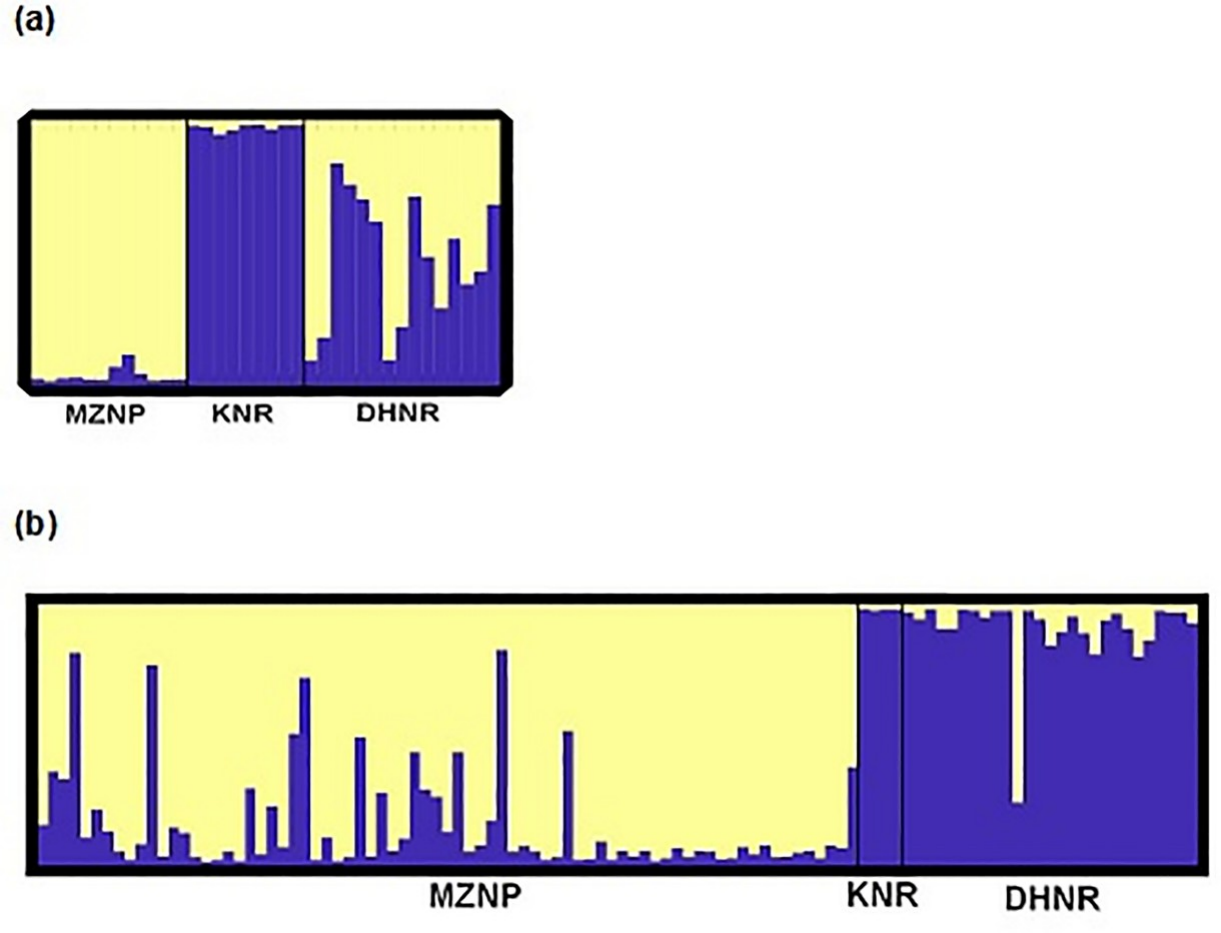
Histogram of multilocus population assignment for three Cape mountain zebra populations. The optimal number of clusters for the dataset was K = 2. Several models were run with very similar results; the admixture model is displayed here. The Mountain zebra National Park (MZNP), De Hoop Nature Reserve (DHNR) and Kammanassie Nature Reserve (KNR) for the two temporal periods, (a) 1999–2001 and (b) 2015–2016.

Principal component analysis (PCA) revealed a clear separation between MZNP and KNR for both the 1999–2001 and 2015–2016 time periods (Fig 2). The position of DHNR was intermediate in the multivariate space between the two relict populations. However, in the 1999–2001 dataset, DHNR appeared closer to the MZNP population, whereas in 2015–2016 it was closer to KNR. In general, populations in 2015–2016 appeared more closely related to each other than in 1999–2001 (Fig 2). Similar results were obtained for STRUCTURE analysis with and without admixture, supporting the occurrence of two distinct genetic clusters (K = 2, Fig 3 and S1 Fig).

In both time periods, MZNP and KNR were assigned to two distinct clusters with high individual coefficient of membership (*qi*) for 1999–2001 MZNP *qi* = 0.9639; 2015–2016 MZNP *q*i = 0.8465 and for the 1999–2001 KNR *qi* = 0.9715; 2015–2016 KNR *q*i = 0.9783. The 1999–2001 DHNR population indicated an approximately 50:50 mixed ancestry with *q*i_MZNP_ = 0.5313 and *q*i_KNR_ = 0.4687 (Fig 3A), while the 2015–2016 DHNR populations were clustered mainly with KNR (*q*i = 0.9048, Fig 3B). Analysis of Molecular Variance (AMOVA) (Table 3) also provided support for variation between the MZNP and KNR populations. For the 1999–2001 time period (MZNP and KNR populations) F_ST_ comparisons indicated that variation between the populations was 54.4% (*p* < 0.001) and variation within populations was 45.58%. In the 2015–2016 time period variation was between MZNP and KNR populations was lower (F_ST_ = 35.35%) with much higher variation within populations (64.65%).

**Table 3:** Results from F_st_-based hierarchical analysis of molecular variance (AMOVA).

Source of variation	Percentage Variation	F_st_	*P*-value
Between 1999–2001 MZNP and KNR populations	54.42	0.544	0.00
Within 1999–2001 MZNP and KNR populations	45.58		
Between 2015–2016 MZNP and KNR	35.35	0.354	0.00
Within 2015–2016 MZNP and KNR populations	64.65		

## Discussion

In this study, we provide a rare test of the genetic consequences of different conservation management strategies among populations of a large mammal. Management recommendations from the BMP included (1) deliberate mixing of relict populations to maintain and improve genetic diversity (excluding KNR and GNR), (2) re-enforcement of existing populations prioritised over the establishment of new populations, (3) translocation of animals to other protected areas, (3) acquisition of land adjacent to protected areas with CMZ, (4) alteration in fire management in the habitat preferred by CMZ to increase availability of palatable grasses; and (5) formation of conservancies with adjacent landowners. However, our data provides support for a general reduction in genetic diversity (A_r_ and H_e_) and loss of private alleles among three key CMZ populations sampled at two time periods (1999–2001 and 2015–2016). These results suggest that despite a high metapopulation growth rate, the CMZ has lost a significant proportion of its genetic diversity within a single generation. At the population level, all three reserves lost genetic diversity (A_r_ and H_e_), with a statistically significant loss being detected in DHNR. The loss of diversity in DHNR was compounded by decreases in H_o_ and an increase in the inbreeding co-efficient (F_IS_). In contrast, non-significant increases in H_o_ and a reduction in F_IS_ were found in the relict populations MZNP and KNR. This slight increase in observed heterozygosity may reflect a sampling effect in KNR, since only four samples were genotyped from this reserve in the 2015–2016 time period. It is more likely due to several loci within these two populations reaching HWE, where H_o_ is not significantly different from H_e_. Both KNR and MZNP, therefore, did not display significant heterozygous excess.

The N_e_ for the MZNP was very low suggesting that continued isolation of this population will result in further loss of genetic variation through drift. A small effective population size could assist in explaining how genetic diversity was lost even though the population demographic census size increased. The effective size can be far smaller than the census size due to a mating system that produces high variance in male and/or female reproductive success. However, bias in this case may be introduced by small sample size, limited time period between collection of samples and number of microsatellite markers [33, 49]. Bias is unlikely to explain entirely the low N_e_ estimates which are well below N_e_ = 50 and can lead to excessive inbreeding and inbreeding depression [50]. Similar low N_e_ values have been reported in other species including red deer (*Cervus elaphus*) from Sardinia and Mesola (N_e_ = 2 to 8) as a consequence of bottlenecks and near-extinction [51].

Of additional concern is the complete loss of private alleles from all three sampled population’s. This suggests rare and low frequency alleles have likely been lost genome wide. While a loss in such population-specific alleles may not have an effect on overall heterozygosity (Norris et al., 1999), it represents a loss of alleles potentially important for future adaptation and loss the uniqueness of the KNR and MZNP stocks. This means that a proportion of the historical diversity of the Cape mountain zebra, conserved through different conservation strategies and present in 1999–2001 has already been lost in the present generation. The loss of unique alleles in MZNP and KNR could also affect an individual or populations ability to adapt and cope with future environmental change. Equally worrying is that the third stock population, inhabiting the Gamkaberg Nature Reserve (GNR), with a growth rate even lower than that of KNR, could also be similarly affected.

The erosion of private alleles may have affected population genetic structure (differentiation) of these populations. Model-based and model-free algorithms all document a decrease in genetic differentiation between the KNR and MZNP stocks, which are now more similar to each other than they were a generation ago. Furthermore, the allele frequencies for DHNR, which were intermediate between its MZNP and KNR founding stocks in 1999–2001, are now clearly more Kammanassie-like, with 90% of genotyped individuals assigned to the KNR cluster (Fig 3). In addition, these results are supported by a reduction in F_ST_ (0.544 to 0.354). Such a substantial shift or homogenization in allele frequencies within a single generation underscores the erosive effect of random genetic drift, even in populations that are expanding demographically, and threatens to undo much of the population benefits of a strategy to restore gene flow.

Major changes in the number of private alleles could also be brought about by non-random mating, where a handful of males dominate most of the breeding opportunities. This idea is corroborated by empirical data showing that in 2005, the population was already male-biased, with a deficit of females resulting in an excess of non-breeding males with limited reproductive potential. Population growth at DHNR has declined from 6.6% in 1995–1999 to 4.5% in 1999–2005 [52]. Thus, DHNR, a population that previously benefitted from admixture, now requires urgent intervention to mitigate this loss. The lower growth rates of KNR and GNR has been attributed to lower abundance of palatable grass species in those reserves [16,53]. The conservative practice of managing these populations separately to protect their uniqueness has had the opposite effect of loss of alleles that made them unique in the first place. Given the evidence of genetic declines reported in this study, the erosive effects of genetic drift and non-random mating can only be rectified through new introductions. Further suggested management practices to facilitate population growth and promote increased genetic diversity include establishing studbooks for all newly founded mixed-stock populations, the use of fertility-control methods to ensure equity in mating opportunities among males and females [54] and ensuring range quality and hence overall body condition on new reserves identified as suitable for CMZ populations. We therefore advocate changes to the conservation management of these important populations, to try to arrest these worrying population trends, likely to cause further loss of diversity, evolutionary potential and the onset of fitness related problems.

Results and the approach from this study could help design and implement management and conservation strategies in other species with only a few small populations remaining. In addition to census size, genetic monitoring of multiple metrics (e.g., heterozygosity, allelic uniqueness, and effective size) can provide early detection of loss of diversity even when a population is large or growing.

## Supporting information

S1 Table Primers used in this study adapted from Horse (*Equus caballus*). (DOCX)

S2 Table Null allele estimations for three Cape mountain zebra populations at two temporal periods. (DOCX)

S3 Table Genetic diversity estimates per marker for three populations; Kammanassie Nature Reserve, Mountain Zebra National Park and DeHoop Nature Reserve over two temporal periods. (DOCX)

S1 Fig Structure Harvester results indicating the ΔK values. (DOCX)

## References

[1] Novellie P, Lindeque M, Lindeque P, Lloyd P, Koen J. Status and Action Plan for the Mountain Zebra (*Equus zebra*), in: Moehlman P. (Ed.), Equids: Zebras, Asses and Horses. IUCN, Gland, Switzerland, p. 204 2002

[2] CITES. Consideration of proposals for amendment of appendicies I and II. Johanneburg. 2016.

[3] Lombard L. Cape Mountain Zebra downlisted at CITES CoP17 [WWW Document]. Traveler24. URL http://traveller24.news24.com/Explore/Green/cape-mountain-zebra-downlisted-at-cites-cop17-20160929, 2016. [Accessed: 22 April 2019]

[4] Milla JCG. Census of Cape Mountain Zebras: Part I. African Wildlife, 1970; 24(1): 16–25.

[5] Millar JCG. Census of Cape Mountain Zebras: Part II. African Wildlife, 1970; 24(2): 104–114.

[6] Hrabar H, Birss C, Peinke D, King S, Kerley G, Child MF. A conservation assessment of *Equus zebra zebra*, in: Child MF, Roxburgh L, Do linh San E, Raimondo D, Davies-Mostert H. (Eds.), The Red List of Mammals of South Africa, Lesotho and Swaziland South African National Biodiversity Institute and Endangered WIldlife Trust, pp. 1–8. 2016.

[7] Moodley Y, Harley EH. Population structuring in mountain zebras (Equus zebra): The molecular consequences of divergent demographic histories. Conserv. Genet. 2005; 6: 953–968. 10.1007/s10592-005-9083-8

[8] Hrabar H, Kerley G. Cape Mountain Zebra 2014/15 Status Report. 2015.

[9] Birss C, Cowell C, Hayward N, Peinke D, Hrabar H, Kotze A. Biodiversity Management Plan for the Cape Mountain Zebra Equus zebra zebra in South Africa, 1–30. 2018.

[10] Penzhorn BL. Habitat selection by Cape mountain zebras in the Mountain Zebra National Park. South African J. Clin. Nutr. 1982; 12: 48–54.

[11] Winkler A, Owen-Smith N. Habitat utilisation by Cape mountain zebras in the Mountain Zebra National Park, South Africa. Koedoe. 1995; 38: 83–93

[12] Lea JM, Kerley GI, Hrabar H, Barry TJ, Shultz S. Recognition and management of ecological refugees: A case study of the Cape mountain zebra. Biol. Conserv. 2016; 203: 207–215. 10.1016/j.biocon.2016.09.017

[13] Marais HJ, Nel P, Bertschinger HJ, Schoeman JP, Zimmerman D. Prevalence and body distribution of sarcoids in South African Cape mountain zebra (*Equus zebra zebra*). J. S. Afr. Vet. Assoc. 2007; 78: 145–148. 10.4102/jsava.v78i3.306 18237037

[14] Sasidharan SP. Sarcoid tumours in Cape mountain zebra (*Equus zebra zebra*) populations in South Africa: a review of associated epidemiology, virology and genetics. Trans. R. Soc. South Africa 2006; 61: 11–18. 10.1080/00359190609519189

[15] Sasidharan SP, Ludwig A, Harper C, Moodley Y, Bertschinger HJ, Guthrie AJ. Comparative Genetics of Sarcoid Tumour-Affected and Non-Affected Mountain Zebra (*Equus zebra*) Populations. South African J. Wildl. Res. 2011; 41: 36–49. 10.3957/056.041.0117

[16] Hill RA. Is isolation the major genetic concern for endangered equids? Anim. Conserv. 2009; 12: 518–519. 10.1111/j.1469-1795.2009.00332.x

[17] Hrabar H, Kerley GIH. Conservation goals for the Cape mountain zebra *Equus zebra zebra*—security in numbers? Oryx 2013; 47: 403–409. 10.1017/S0030605311002018

[18] Watson LH, Odendaal HE, Barry TJ, Pietersen J. Population viability of Cape mountain zebra in Gamka Mountain Nature Reserve, South Africa: The influence of habitat and fire. Biol. Conserv. 2005; 122: 173–180. 10.1016/j.biocon.2004.06.014

[19] Watson LH, Chadwick P. Management of Cape mountain zebra in the Kammanassie Nature Reserve, South Africa. South African J. Wildl. Res. 2007; 37: 31–39. 10.3957/0379-4369-37.1.31

[20] Moodley Y. Population structuring in southern African Zebras. University of CapeTown. 2002.

[21] Van Oosterhout C, Hutchinson WF, Wills DPM, Shipley P. MICRO-CHECKER: Software for identifying and correcting genotyping errors in microsatellite data. Mol. Ecol. Notes 2004; 4: 535–538. 10.1111/j.1471-8286.2004.00684.x

[22] Kallinowski ST. HP-Rare: A computer program for performing rarefications on measures of allelic diversity. Molecular Ecology Notes 2005; 5:187–189

[23] Peakall R, Smouse PE. genalex 6: genetic analysis in Excel. Population genetic software for teaching and research. Mol. Ecol. Notes 2005; 6; 288–295. 10.1111/j.1471-8286.2005.01155.x PMC3463245 22820204

[24] Peakall R, Smouse PE. GenALEx 6.5: Genetic analysis in Excel. Population genetic software for teaching and research-an update. Bioinformatics 2012; 28: 2537–2539. 10.1093/bioinformatics/bts460 22820204 PMC3463245

[25] Guo SW, Thompson EA. Performing the Exact Test of Hardy-Weinberg Proportion for Multiple Alleles. Biometrics 1992; 48: 361 10.2307/2532296 1637966

[26] Piry S, Luikart G, Cornuet J-M. BOTTLENECK: a program for detecting recent effective population size reductions from allele data frequencies. J. Hered. 1999; 90: 502–503. 10.1093/jhered/90.4.502

[27] Cornuet JM, Luikart G. Description and power analysis of two tests for detecting recent population bottlenecks from allele frequency data. Genetics 1996; 144: 2001–2014. 10.12968/vetn.2014.5.7.372 8978083 PMC1207747

[28] Di Rienzo A, Peterson AC, Garza JC, Valdes AM, Slatkin M, Freimer NB. Mutational processes of simple-sequence repeat loci in human populations. Proc. Natl. Acad. Sci. 1994; 91: 3166–3170. 10.1073/pnas.91.8.3166 8159720 PMC43536

[29] Luikart G, Cornuet JM. Empirical evaluation of a test for identifying recently bottlenecked populations from allele frequency data. Conserv. Biol. 1998; 12: 228–237. 10.1046/j.1523-1739.1998.96388.x

[30] Luikart G, Sherwin WB, Steele BM, Allendorf FW. Usefulness of molecular markers for detecting population bottlenecks via monitoring genetic change. Mol. Ecol. 1998; 7: 963–974. 10.1046/j.1365-294x.1998.00414.x 9711862

[31] Luikart G, Allendorf FW, Cornuet JM, Sherwin WB. Distortion of allele frequency distributions provides a test for recent population bottlenecks. J. Hered. 1998; 89: 238–247. 10.1093/jhered/89.3.238 9656466

[32] Krimbas CB, Tsakas S. The Genetics of *Dacus oleae*. V. changes of esterase polymorphism in a natural population following insecticide control- Selection or drift. Evolution (N. Y). 1971; 25: 454–460. 10.1111/j.1558-5646.1971.tb01904.x 28565021

[33] Nei M, Tajima F. Genetic drift and estimation of effective population size. Genetics 1981; 98: 625–640. 10.1016/j.pain.2014.03.008 17249104 PMC1214463

[34] Waples RS. A generalized Approach for Estimating Effective Population Size from Temporal Changes in Allele Frequency. Genetics. 1989; 121:379–91. 2731727 10.1093/genetics/121.2.379 PMC1203625

[35] Fisher RA. The genetical theory of natural selection. 272pp. Oxford: Clarendon press. 1930.

[36] Wright S. Evolution in Mendelian populations. Genetics. 1931; 16:97–159. 17246615 10.1093/genetics/16.2.97 PMC1201091

[37] Jorde PE, Ryman N. Unbiased estimator for genetic drift and effective population size. Genetics 2007; 177: 927–935. 10.1534/genetics.107.075481 17720927 PMC2034655

[38] Do C, Waples RS, Peel D, Macbeth GM, Tillett BJ, Ovenden JR. NeEstimator v2: Re-implementation of software for the estimation of contemporary effective population size (Ne) from genetic data. Mol. Ecol. Resour. 2014; 14: 209–214. 10.1111/1755-0998.12157 23992227

[39] Jombart T. adegenet: a R package for the multivariate analysis of genetic markers. Bioinformatics 2008; 24:1403–1405. 10.1093/bioinformatics/btn129 18397895

[40] Pritchard JK, Stephens M, Donnelly P. Inference of population structure using multilocus genotype data. Genetics 2000; 155:945–959. 10.1111/j.1471-8286.2007.01758.x 10835412 PMC1461096

[41] Evanno G, Regnaut S, Goudet J. Detecting the number of clusters of individuals using the software structure: a simulation study. Mol. Ecol. 2005; 14: 2611–2620. 10.1111/j.1365-294X.2005.02553.x 15969739

[42] Earl DA, VonHoldt BM. STRUCTURE HARVESTER: A website and program for visualizing STRUCTURE output and implementing the Evanno method. Conserv. Genet. Resour. 2012; 4: 359–361. 10.1007/s12686-011-9548-7

[43] Jakobsson M, Rosenberg NA. CLUMPP: A cluster matching and permutation program for dealing with label switching and multimodality in analysis of population structure. Bioinformatics 2007; 23: 1801–1806. 10.1093/bioinformatics/btm233 17485429

[44] Rosenberg NA. distruct: a program for the graphical display of population structure. Mol. Ecol. Notes 2003; 4: 137–138. 10.1046/j.1471-8286.2003.00566.x

[45] Excoffier L, Smouse PE, Quattro JM. Analysis of molecylar Varriance Inferred From Metric Distances Among DNA Haplotypes: Applicatio to Human Mitochondrial DNA Restriction Data. Genetics 1992; 131: 479–491. 1644282 10.1093/genetics/131.2.479 PMC1205020

[46] Excoffier L., Lischer H.E.L. Arlequin suite ver 3.5: A new series of programs to perform population genetics analyses under Linux and Windows. Mol. Ecol. Resour. 2010; 10: 564–567. 10.1111/j.1755-0998.2010.02847.x 21565059

[47] Rice WR. Analyzing tables of statistical tests. H. Am. Sociol. Rev. 1989; 78: 1–3. 10.1111/j.0014-3820.2001.tb00731.x 28568501

[48] Lorenzen ED, Arctander P, Siegismund HR. High variation and very low differentiation in wide ranging plains zebra (*Equus quagga*): insights from mtDNA and microsatellites. Mol. Ecol. 2008; 17: 2812–2824. 10.1111/j.1365-294X.2008.03781.x 18466230

[49] Waples RS, Yokota M. Temporal estimates of effective population size in species with overlapping generations. Genetics 2007; 175: 219–233. 10.1534/genetics.106.065300 17110487 PMC1775005

[50] Jamieson IG, Allendorf FW. How does the 50/500 rule apply to MVPs? Trends Ecol Evol. 2012;27(10):580–4. Available from: 10.1016/j.tree.2012.07.001 22868005

[51] Zachos FE, Frantz AC, Kuehn R, Bertouille S, Colyn M, Niedziałkowska M, et al Genetic Structure and Effective Population Sizes in European Red Deer (*Cervus elaphus*) at a Continental Scale: Insights from Microsatellite DNA. J. Hered. 2016; 107: 318–326. 10.1093/jhered/esw011 26912909 PMC4888435

[52] Smith RK, Marais A, Chadwick P, Lloyd PH, Hill RA. Monitoring and management of the endangered Cape mountain zebra *Equus zebra zebra* in the Western Cape, South Africa. Afr. J. Ecol. 2007; 46: 207–213. 10.1111/j.1365-2028.2007.00893.x

[53] Vlok JHJ, Cowling RM, Wolf T. A vegetation map for the Little Karoo. Unpublished maps and report for a SKEP project supported by Grant No 1064410304. (Cape Town, Critical Ecosystem Partnership Fund) 2005.

[54] National Research Council, 2013 Using science to improve the BLM Wild Horse and Burro Program: a way forward. National Academies Press.

