# Supplementary material for: Lessons for conservation management: Monitoring temporal changes in genetic diversity of Cape mountain zebra (Equus zebra zebra)
Source: PLoS One. 2019 Jul 31;14(7):e0220331. doi: 10.1371/journal.pone.0220331 (PMC6668792; doi:10.1371/journal.pone.0220331)
Supplement: S1 Table — (DOCX) [file pone.0220331.s001.docx]

**S1 Table.** **Primers used in this study adapted from Horse (*Equus caballus*).** T(a) indicates annealing temperature.

| Locus | Forward primer sequence (5’-3’) | Reverse primer sequence 5’-3’ | T(a) | Repeat |
| --- | --- | --- | --- | --- |
| HMB1 | GTGTGTATGCTTCCCAACCCTT | GTTATAAAGCACTATGATCTCA | 58 | (GT)_18_ |
| HTG 14 | CCAGTCTAAGTTTGTTGGCTAGAA | CAAAGGTGAGTGATGGATGGAAGC | 55 | (TG)_14_ |
| HTG09 | TGTGGGAAGAGTGTCAATAGCTGT | AGGCATCTGGTTTGCTGCAATTTC | 55 | (GT)_17_ |
| HTG11 | CAATGATGGTACTTTGCATATTAA | ATCGGCATGCACACTCATAGGTAG | 59 | (GT)_15_ |
| HTG15 | TCTTGATGGCAGAGCCAGGATTTG | AATGTCACCATGCGGCACATGACT | 55 | (TG)_14_ |
| HTG7 | CCTGAAGCAGAACATCCCTCCTTG | ATAAAGTGTCTGGGCAGAGCTGCT | 55 | (TG)_19_ |
| LEX20 | GGAATAGGTGGGGGTCTGTT | AGGGTACTAGCCAAGTGACTGC | 55 | (TG)_19_ |
| LEX52 | GGAACGGAAGAGTGTAGTTTT | CATTTATTCATCAGCGATTTG | 55 | (TG)_13_ |
| TKY273 | GATCACTGGCGAGGGTAAGC | TATGTTCCCGATTCGCAAGC | 55 | (CA)_22_ |
| UCDEQ505 | ATCACTCTCTTGTTGAGATAAC | GGGATTTCCTTCTTTCTC | 55 | (GT)_17_ |
| VHL47 | GTTTGCTGTGGTTACCAGGCAGA | GCAAATTGAATATTTGAAGTTGAGAC | 55 | (CA)_13_ |
| COR014 | CTATCATGTCAGGGACCAGG | CTGCCCTAGTTAGCAACCAA | 58 | (GT)_21_ |
| AHT21 | TCCAAGTTGCTGAATGGATC | ACGGCCTGATTCTCTCTTTG | 60 | (GT)_21_ |
| HTG3 | TAACCTGGGTGCAAAGCCACCCAT | GTCAGGGCCAATCTTCCTCAC | 63 | (TG)_16_ |
| HTG5 | TGCTAAGCCTCAGCACATACA | TGGAAATAAGGTTAGCAGGGATGC | 63 | (TG)_15_ |
