# Supplementary material for: Lessons for conservation management: Monitoring temporal changes in genetic diversity of Cape mountain zebra (Equus zebra zebra)
Source: PLoS One. 2019 Jul 31;14(7):e0220331. doi: 10.1371/journal.pone.0220331 (PMC6668792; doi:10.1371/journal.pone.0220331)
Supplement: S2 Table — (DOCX) [file pone.0220331.s002.docx]

| **S2 Table.** **Null allele estimations for three Cape mountain zebra populations at two temporal periods.** The data was tested using the methods from Oosterhout and Chakraborty. | | | | | | | |
| --- | --- | --- | --- | --- | --- | --- | --- |
| **Mountain zebra National Park** | | | | | | | |
| **1999-2001** | | | | **2015-2016** | | | |
| **Locus** | **Null Present** | **Oosterhout** | **Chakraborty** | **Locus** | **Null Present** | **Oosterhout** | **Chakraborty** |
| HTG07 | no | 0.1104 | 0.122 | HTG07 | no | -0.0341 | -0.0351 |
| HTG15 | no | 0 | 0 | HTG15 | no | 0 | 0 |
| HMB1 | no | 0.0787 | 0.0795 | HMB1 | no | -0.0216 | -0.024 |
| LEX20 | no | 0.1619 | 0.1538 | LEX20 | no | -0.015 | -0.0075 |
| VHL47 | no | 0 | 0 | VHL47 | no | 0.1016 | 0.1527 |
| UCDEQ505 | no | 0 | 0 | UCDEQ505 | no | -0.0426 | -0.0382 |
| **COR014** | **yes** | **0.3416** | **1** | **COR014** | **yes** | **0.1024** | **0.1603** |
| LEX52 | no | 0 | 0 | LEX52 | no | 0 | 0 |
| AHT21 | no | 0 | 0 | AHT21 | no | 0.0051 | -0.0096 |
| TKY273 | no | 0 | 0 | TKY273 | no | -0.0134 | -0.0067 |
| HTG14 | no | 0.1469 | 0.1111 | HTG14 | no | 0.0888 | 0.2338 |
| HTG03 | no | 0 | 0 | HTG03 | no | 0 | 0 |
| HTG9 | no | 0.1142 | 0.1416 | HTG9 | no | 0.0343 | 0.04 |
| HTG11 | no | 0.2296 | 0.4286 | HTG11 | no | 0.038 | 0.047 |
|  |  |  |  |  |  |  |  |
| **De Hoop Nature Reserve** | | | | | | | |
| **1999-2001** | | | | **2015-2016** | | | |
| **Locus** | **Null Present** | **Oosterhout** | **Chakraborty** | **Locus** | **Null Present** | **Oosterhout** | **Chakraborty** |
|  |  |  |  |  |  |  |  |
| HTG07 | no | 0.0922 | 0.0947 | HTG07 | no | 0.1041 | 0.1099 |
| HTG15 | no | 0 | 0 | HTG15 | no | 0 | 0 |
| HMB1 | no | 0.1945 | 0.4667 | HMB1 | no | 0 | 0 |
| LEX20 | no | -0.3767 | -0.2329 | LEX20 | no | -0.077 | -0.0385 |
| VHL47 | no | -0.1056 | -0.0526 | VHL47 | no | -0.077 | -0.0385 |
| UCDEQ505 | no | -0.1162 | -0.0959 | **UCDEQ505** | **yes** | **0.3411** | **0.6981** |
| COR014 | no | -0.0531 | -0.049 | COR014 | no | 0.1826 | 0.25 |
| LEX52 | no | 0 | 0 | LEX52 | no | 0 | 0 |
| AHT21 | no | -0.0259 | -0.0365 | AHT21 | no | -0.0388 | -0.0371 |
| TKY273 | no | 0.0246 | 0.0255 | TKY273 | no | -0.2404 | -0.1183 |
| HTG14 | no | -0.0364 | -0.0345 | HTG14 | no | -0.0889 | -0.0781 |
| HTG03 | no | 0 | 0 | HTG03 | no | 0 | 0 |
| HTG9 | no | -0.1093 | -0.0909 | HTG9 | no | -0.3118 | -0.1321 |
| HTG11 | no | 0.1547 | 0.25 | HTG11 | no | -0.0187 | -0.0093 |
|  |  |  |  |  |  |  |  |

| **Kamanassie Nature Reserve** | | | | | | | |
| --- | --- | --- | --- | --- | --- | --- | --- |
| **1999-2001** | | | | **2015-2016** | | | |
| **Locus** | **Null Present** | **Oosterhout** | **Chakraborty** | **Locus** | **Null Present** | **Oosterhout** | **Chakraborty** |
|  |  |  |  |  |  |  |  |
| HTG07 | no | 0.1046 | 0.1045 |  | | | |
| HTG15 | no | 0 | 0 |  |  |  |  |
| HMB1 | no | 0.0787 | 0.0924 |  |  |  |  |
| LEX20 | no | -0.1835 | -0.0909 |  |  |  |  |
| VHL47 | no | -0.1181 | -0.0588 |  |  |  |  |
| UCDEQ505 | no | 0 | 0 |  | Insufficient sample size | |  |
| COR014 | no | 0 | 0 |  |  |  |  |
| LEX52 | no | 0 | 0 |  |  |  |  |
| AHT21 | no | 0 | 0 |  |  |  |  |
| TKY273 | no | 0.1498 | 0.2174 |  |  |  |  |
| HTG14 | no | -0.3333 | -0.1613 |  |  |  |  |
| HTG03 | no | 0 | 0 |  |  |  |  |
| HTG9 | no | 0 | 0 |  |  |  |  |
| HTG11 | no | 0.3797 | 1 |  |  |  |  |
|  |  |  |  |  |  |  |  |
