# Supplementary material for: Lessons for conservation management: Monitoring temporal changes in genetic diversity of Cape mountain zebra (Equus zebra zebra)
Source: PLoS One. 2019 Jul 31;14(7):e0220331. doi: 10.1371/journal.pone.0220331 (PMC6668792; doi:10.1371/journal.pone.0220331)
Supplement: S3 Table — (DOCX) [file pone.0220331.s003.docx]

**S3 Table. Genetic diversity estimates per marker for three populations; Kammanassie Nature Reserve, Mountain Zebra National Park and DeHoop Nature Reserve over two temporal periods.** A_r_ = the Allelic richness for which is corrected for sample size, A_e_ = number of effective alleles, I = Shannon's information index, H_o_ = Observed heterozygosity, H_e_ = expected heterozygosity, F = fixation index, HWE = deviation from Hardy-Weinberg Equilibrium (* = Significant value p < 0.05).

| **Population** | **Locus** | **A_r_** | **A_e_** | **I** | **H_o_** | **H_e_** | **F** | **HWE** |
| --- | --- | --- | --- | --- | --- | --- | --- | --- |
| 1999-2001 | HTG07 | 3.2112 | 3.574 | 1.483 | 0.5313 | 0.732 | 0.262 | 0.0142 |
|  | HMB1 | 2.4141 | 2.481 | 1.117 | 0.2647 | 0.606 | 0.557 | **0.0000*** |
|  | LEX20 | 2.1543 | 1.755 | 0.766 | 0.4857 | 0.436 | -0.129 | 0.0545 |
|  | VHL47 | 1.4668 | 1.635 | 0.577 | 0.1389 | 0.394 | 0.643 | **0.0003*** |
|  | UCDEQ505 | 16663 | 2.190 | 0.850 | 0.3125 | 0.552 | 0.425 | **0.0127*** |
|  | COR014 | 1.6656 | 2.253 | 0.883 | 0.2188 | 0.565 | 0.607 | **0.0000*** |
|  | AHT21 | 1.9428 | 2.034 | 0.812 | 0.4063 | 0.516 | 0.201 | 0.2466 |
|  | TKY273 | 1.5575 | 1.619 | 0.571 | 0.2727 | 0.388 | 0.287 | 0.1561 |
|  | HTG14 | 2.4252 | 2.187 | 0.897 | 0.4722 | 0.550 | 0.130 | 0.0737 |
|  | HTG9 | 2 | 2.033 | 0.862 | 0.3824 | 0.516 | 0.248 | **0.0017*** |
|  | HTG11 | 2.2631 | 1.552 | 0.662 | 0.1154 | 0.363 | 0.676 | **0.0001*** |
|  |  | **2.069** | **2.120** | **0.862** | **0.327** | **0.511** | **0.355** |  |
| 2015-2016 | HTG07 | 2.9511 | 2.679 | 1.091 | 0.621 | 0.630 | 0.009 | 0.1494 |
|  | HMB1 | 1.756 | 2.175 | 0.842 | 0.353 | 0.543 | 0.347 | **0.0000*** |
|  | LEX20 | 1.3707 | 1.063 | 0.137 | 0.061 | 0.060 | -0.032 | 1.0000 |
|  | VHL47 | 1.5868 | 1.227 | 0.331 | 0.147 | 0.186 | 0.204 | 0.0688 |
|  | UCDEQ505 | 1.6557 | 1.679 | 0.594 | 0.271 | 0.406 | 0.330 | **0.0022*** |
|  | COR014 | 1.6625 | 1.540 | 0.571 | 0.188 | 0.352 | 0.463 | **0.0000*** |
|  | AHT21 | 2.3081 | 1.739 | 0.730 | 0.390 | 0.427 | 0.081 | **0.0043*** |
|  | TKY273 | 15594 | 1.142 | 0.245 | 0.133 | 0.125 | -0.071 | 1.0000 |
|  | HTG14 | 1.7986 | 1.418 | 0.471 | 0.184 | 0.296 | 0.374 | **0.0005*** |
|  | HTG9 | 2.4241 | 2.353 | 0.956 | 0.554 | 0.578 | 0.036 | 0.3008 |
|  | HTG11 | 1.3906 | 1.135 | 0.237 | 0.108 | 0.120 | 0.096 | 0.3348 |
|  |  | **1.8603** | **1.650** | **0.564** | **0.274** | **0.338** | **0.167** |  |
