# Supplementary material for: Lessons for conservation management: Monitoring temporal changes in genetic diversity of Cape mountain zebra (Equus zebra zebra)
Source: PLoS One. 2019 Jul 31;14(7):e0220331. doi: 10.1371/journal.pone.0220331 (PMC6668792; doi:10.1371/journal.pone.0220331)
Supplement: S1 Fig — (DOCX) [file pone.0220331.s004.docx]

| (a)   | (b)   |
| --- | --- |
| (c)   | (d)   |

**S1 Fig.** **Structure Harvester results indicating the ΔK values (a and c) and log-likelihood (b and d) values which suggest the most likely K-value for among three Cape mountain zebra populations, MZNP, DHNR and KNR for the periods 1999-2001 (a) and (b) and 2015-2016 (c) and (d).**
